# Total knee replacement in Osteogenesis Imperfecta: a case report and review of the literature

**DOI:** 10.1186/s42836-020-00061-5

**Published:** 2021-04-02

**Authors:** Allan Roy Sekeitto, Kaeriann van der Jagt, Nkhodiseni Sikhauli, Dick Ronald van der Jagt

**Affiliations:** Arthroplasty Unit, Division of Orthopaedic Surgery, Charlotte Maxeke Johannesburg Academic Hospital, University of the Witwatersrand, 7 York Road, Parktown, Johannesburg, South Africa

**Keywords:** Osteogenesis imperfecta, Total knee replacement, Arthroplasty, Hinged prosthesis

## Abstract

**Background:**

A review of the literature revealed that only 9 total knee replacements were performed on patients with osteogenesis imperfecta (OI), with one being a revision procedure of a periprosthetic fracture. Of the 9 primary procedures, all used cemented prostheses, and 3 patients had an osteotomy at the same procedure. Our patient required a hinged prosthesis because of collateral ligament incompetence and is the first such case reported in the literature.

**Case presentation:**

Presented here is a total knee replacement performed on a 52-year-old patient with osteogenesis imperfecta (OI) who injured her left knee and ruptured her anterior cruciate ligament. Her right knee suffered from severe degenerative changes with an incompetent medial collateral ligament. It was decided to replace the right knee before addressing the left knee injury. A hinged revision prosthesis was used. The smallest components available were used because of the small anatomical bony dimensions.

**Conclusion:**

This is the first reported case of a hinged prosthesis and highlights the soft tissue component of osteogenesis imperfecta. We also highlight the technical problems with these patients, including mal-alignment, small bony dimensions and bone fragility.

## Background

Osteogenesis Imperfecta (OI) is a rare congenital disorder that affects connective tissue. It presents in 1 in 20,000 births and is caused by a genetic alteration resulting in abnormal collagen structure, ossification and mineralisation. This condition leads to various orthopaedic manifestations and inherent coagulation defects due to abnormal collagen within the endothelial capillary bed [1, 2].

Amongst the described orthopaedic manifestations are protrusio acetabuli, trefoil pelvis, ligament laxity, osteoporosis, fragile bone, malunion, non-union and limb deformities. The distortion of the normal anatomy with significance to a total knee arthroplasty is the femur that often has a shepherd-crook’s deformity [3]. OI patients, thanks to modern medical advances, have a virtually normal life span [4]. The joint laxity and fractures with secondary joint degeneration leads to hip and knee osteoarthrosis requiring joint arthroplasty [1].

Patients present with a broad spectrum of severity, ranging from intrauterine death to milder forms only diagnosed later in adulthood. The long bones are prone to bowing, fracture, subsequent malunion and non-union, with associated narrowing of the intramedullary canal. Adults present with early severe arthritis, long bone deformities and scoliosis [5].

Here we present a case study of a patient with OI who underwent a total knee arthroplasty.

## Case report

**Fig. 1:**
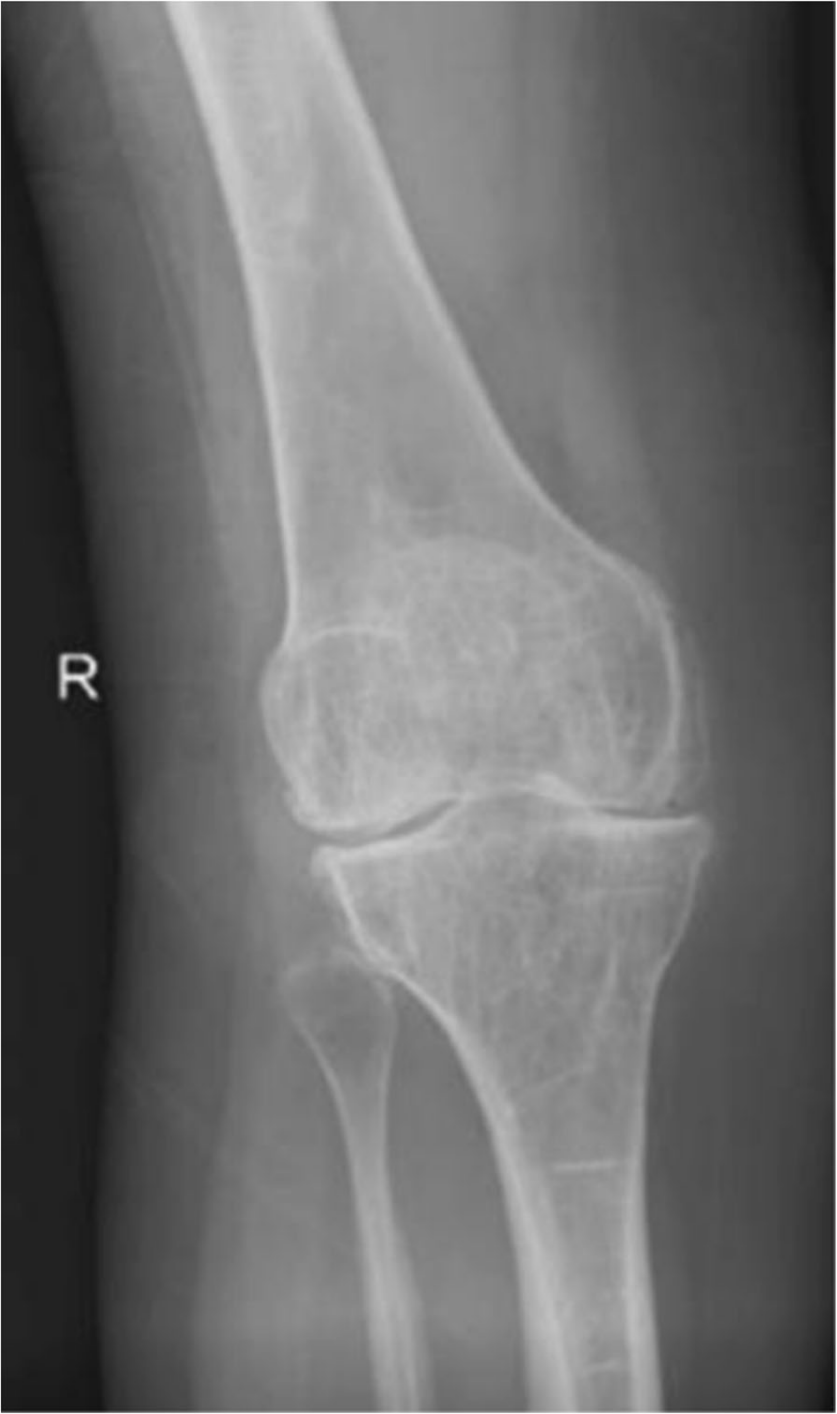
A preoperative AP radiograph demonstrating the degenerative changes in the knee

**Fig. 2:**
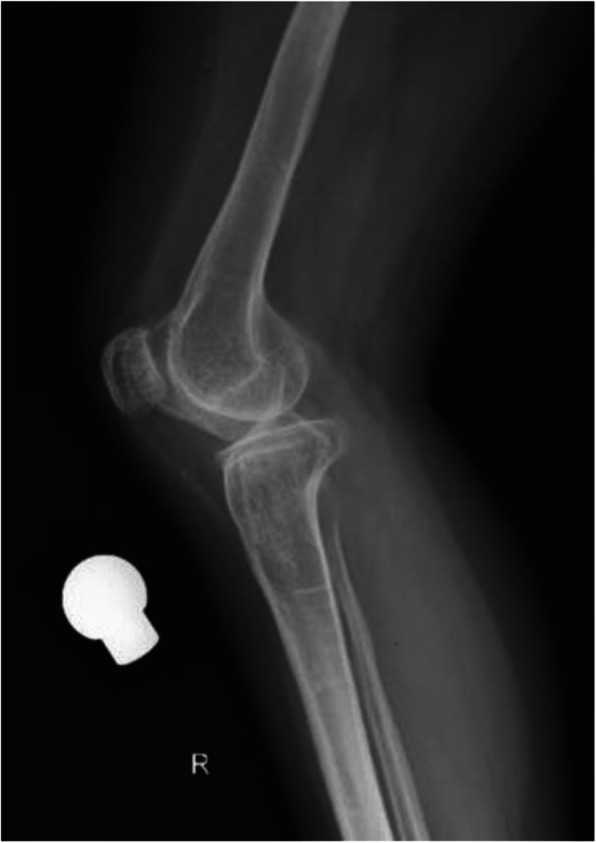
A preoperative lateral radiograph demonstrating the degenerative changes in the knee

A 52-year-old female patient with OI had recently injured her left knee, rupturing her anterior cruciate ligament (ACL). She had pre-existing degenerative changes in her right knee with a deficient medical collateral ligament (MCL) of clinical grade 3, which had been symptomatic for 2 years prior to her fall (Figs. 1 and 2). We opted to perform a right total knee replacement before addressing the left knee injury. This would provide her with an immediate stable right limb prior to addressing her left knee.

**Fig. 3:**
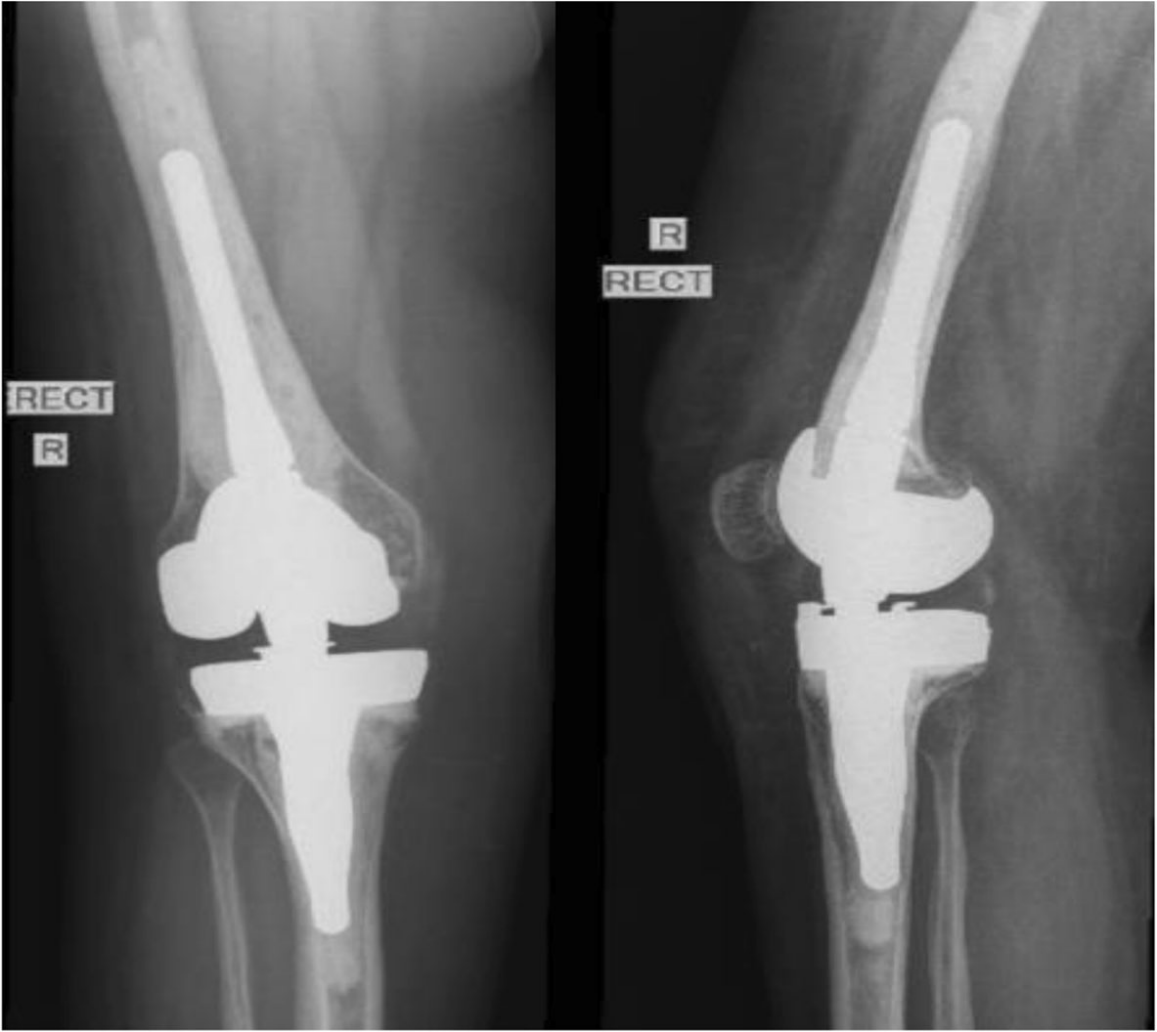
A postoperative AP and lateral radiograph of the knee demonstrating the cemented hinged prosthesis

A Nextgen Rotating Hinge Knee (ZimmerBiomet®) hinged prosthesis was used because of her incompetent MCL (Fig. 3). The smallest components available were used because of her small anatomical bony dimensions. The OI population tend to experience growth retardation and standard implant sizes are generally over-sized for these patients. This necessitates pre-operative planning in the form of templating to ensure the specific implant sizes are available for the patient. In the absence of such implants, patient-specific implants and cutting guides should be considered for outlier cases [5]. The initial postoperative course was uneventful. The patient mobilised slowly though. She had poor flexion at 6 weeks and a gentle manipulation was performed cognizant of the inherent dangers. She is currently at 2-year post-op with full extension and flexion to 110 degrees. The patient-reported outcome measures improved as follows: her visual analogue score (VAS) was 7/10, 3/10 and 2/10 before, 6 and 24 months after surgery respectively. The Western Ontario and McMaster Universities Osteoarthritis Index (WOMAC) knee score was 36.4, 59.4 and 86.4 before, 6 and 24 months after surgery respectively. She continued to wear a brace on left knee as she was reluctant to undergo an ACL reconstruction.

## Discussion

A review of the literature revealed that only 9 total knee replacements were performed on OI patients (Table 1). The 9 primary procedures, all employed cemented prostheses, and 3 patients needed an osteotomy at the same procedure. Two cases received revision components, and one was due to an intra-operative periprosthetic fracture that necessitated the revision of the tibial component to a stemmed component with augments during the primary procedure. With the second case, the revision was performed with a total femur component and a stemmed tibial component due to a failed hip femoral component, and this failure is due to perforation through the trochlea. The constraint of the prosthesis in the literature is shown in Table 1. Five posterior stabilised, three cruciate retaining and a single hinge implants were used in the total knee replacements. All the procedures were indicated for secondary osteoarthritis except the single case of a total femur replacement with a hinged prosthesis, with the patient having developed a complication related to a previous failure of hip femur revision. The average age was 50 years, which was similar to the age of our patient. The case studies reported good pain relief and improved functional outcomes in the patients that underwent total knee replacements [4, 5].

**Table 1:** Published articles with total knee replacements for Osteogenesis Imperfecta

Article	No. of patients	Age	Indication	Implants
Papagelopoulos *et al*. [4]	2	54, F	Bilateral Osteoarthritis	Posterior-stabilised ×2
		46, F	Osteoarthritis	Cruciate-retaining
Nishimura *et al*. [6]	1	53, M	Osteoarthritis	Cruciate-retaining
Wagner *et al*. [5]	2	46, M	Osteoarthritis	Cruciate-retaining, femur and tibia osteotomies (femur retrograde intramedullary nail and flexible titanium intramedullary nails)
		37, M	Bilateral Osteoarthritis	**Right**: Posterior-stabilised, femur and tibia osteotomies (flexible titanium intramedullary nails) **Left:** Posterior-stabilised, femur and tibia osteotomies (flexible titanium intramedullary nails and dynamic compression plates) **Left Conversion**: tibia intraoperative periprosthetic fracture, tibia component revised cemented tibia 80 mm stem and 10 mm augment during the primary procedure
Brand *et al*. [7]	1	69, F	Osteoarthritis	Posterior-stabilised
Sanz-Ruiz *et al*. [1]	1	65, F	Hip femoral stem perforated through trochlea	Total femur replacement, hinged prosthesis and stemmed tibia

### Component sizing

Nishimura and colleagues [6] presented a case for whom they had planned to use posterior constrained components for the total knee replacement. They found the posterior stabilised components was too large for their patient and had to switch to a cruciate retaining prosthesis. This was similar to our case where the smallest components were used [4]. In our case, we used the smallest components available to perform the total knee replacement. The patient population with OI tend to experience growth retardation and standard implant sizes are generally found to be too big for these patients. This necessitates meticulous pre-operative planning in the form of templating using sizing markers on the planning radiographs to ensure the specific implant size is available for the patient. In the absence of such implants, patient-specific implants and cutting guides should be considered for outlier cases [7].

### Hinged- prothesis

Our case study is the first in the literature to describe a case of a primary total knee replacement performed with a hinged prosthesis for OI-related osteoarthritis. Sanz-Ruiz *et al*. presented a case of a total femur replacement and hinged knee with a stemmed tibia secondary to a hip femoral revision component that failed and perforated the femoral trochlea (1). This was due to the poor bone quality in OI patients. The generalised laxity presents another challenge to total knee arthroplasty in obtaining a stable knee postoperatively. In patients with severe coronal deformities, the soft tissues are lax, depending on whether the convexity lies on the medial or lateral side and this may result in such tissue imbalance. The milder soft tissue imbalances can be managed with soft tissue release intraoperatively. However, constrained implants should be considered for the deformities of more than 20 degrees [7, 8]. In our case, we performed a total knee replacement with a hinged prosthesis due to the patient’s incompetent medial collateral ligament.

### Long bone deformities

Long bone bowing and the resultant deformities pose an alignment challenge and the mechanical axis and angular deformities must be evaluated preoperatively [7]. The femur presents with 46–86% anterolateral bowing and associated 27–86% anterior bowing of the tibia [4].

The management options are either an extra-articular osteotomy [5] or an adjusted intra-articular resection [4]. These procedures can be proposed at the time before total knee placement or as a staged procedure. The intra-articular resection has the advantage of reducing second site complications. Nonetheless, this modality is limited by the extent of the presenting deformity. In their series of three total knee replacements with concurrent osteotomies, Wagner *et al*. [5], had a single case of tibia non-union 11.5 months after the procedure, which was managed with open reduction and internal fixation and augmented with iliac crest autograft. The use of intra-medullary referencing in patients with deformity or a narrow canal with deformity may lead to iatrogenic fractures and recommendations have been made for extramedullary referencing techniques, patient-specific cutting blocks or navigation assistance [6, 7].

### Poor bone quality

The poor bone quality results in complications. Wagner and colleagues [5] reported intraoperative fractures in their series, including a non-displaced fracture of the patella and a compression fracture of the proximal tibia that required revision to a stemmed tibial component with augments. The patient should be handled carefully especially during trialing and definitive component placement to prevent iatrogenic fractures [7]. An extension stem of the tibial component could help address osteopenia in the metaphysis [7]. The osteopenia may lead to thinned patellae, which can fracture and therefore the literature recommends against patella resurfacing [6, 7]. A single case of patella fracture was reported in the literature [5]. In our case, we did not resurface the patella.

### Anaesthesia and theatre set up

The literature recommends that during theatre settingup, a well-padded operating table should be used to the protect the patient from injury [7] since deformities, common in these patients, can easily lead to pressure sores. Regional anesthesia is preferred but is often contraindicated because of anatomical anomalies such as scoliosis. In cases where general anaesthesia is elected, fiberoptic endoscopy-aided intubation was used to avoid cervical spine fractures or injury due to instability which should be screened for preoperatively [4, 7]. The patients are at risk of chipping of teeth, mandibular fractures or may have airway abnormalities secondary to a large tongue or a short neck [4]. In our case, the patient had a spinal anaesthesia supplemented by a general anaesthetic.

## Conclusion

There is a paucity of literature on total knee replacement in OI patients. The improved medical care has improved patients' life span. We foresee an increase in the demand for arthroplasty in this population. The importance of understanding the underlying disease, component sizing, adequate constraint, management of long bone deformities, mitigation of iatrogenic injury and anaesthetic risk must be addressed for a safe surgery and a satisfactory outcome. Total knee replacement should be part of the long term management of OI patients.

## Data Availability

The datasets used and analysed during the current study are available from the corresponding author on reasonable request.

## Abbreviations

ACL: Anterior cruciate ligament
MCL: Medical collateral ligament
OI: Osteogenesis imperfecta
