# Correction to: Total knee replacement in Osteogenesis Imperfecta: a case report and review of the literature

**DOI:** 10.1186/s42836-021-00079-3

**Published:** 2021-05-11

**Authors:** Allan Roy Sekeitto, Kaeriann van der Jagt, Nkhodiseni Sikhauli, Dick Ronald van der Jagt

**Affiliations:** Arthroplasty Unit, Division of Orthopaedic Surgery, Charlotte Maxeke Johannesburg Academic Hospital, University of the Witwatersrand, 7 York Road, Parktown, Johannesburg, South Africa

**Correction to: Arthroplasty 3, 4 (2021)**

**https://doi.org/10.1186/s42836-020-00061-5**

Following publication of the original article [1], the authors reported an error for some numbers in the sentence “her visual analogue score (VAS) was 3/10, 7/10 and 8/10 before, 6 and 24 months after surgery respectively” in the Case report section.

The numbers should be corrected into “7/10, 3/10 and 2/10”.

The original article [1] has been updated.

## References

[1] Sekeitto AR, van der Jagt K, Sikhauli N, van der Jagt DR (2021). Total knee replacement in Osteogenesis Imperfecta: a case report and review of the literature. Arthroplasty.

